# Supplementary material for: Map plasticity following noise exposure in auditory cortex of rats: implications for disentangling neural correlates of tinnitus and hyperacusis
Source: Front Neurosci. 2024 May 31;18:1385942. doi: 10.3389/fnins.2024.1385942 (PMC11176560; doi:10.3389/fnins.2024.1385942)
Supplement: Supplementary file 1 [file Data_Sheet_1.docx]

Supplementary Material

# Supplementary Figures

**
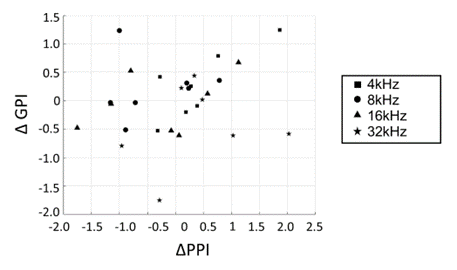
**

**Supplementary Figure S1.** Behavioral metrics of hyperacusis (ΔPPI) vs. tinnitus (ΔGPI).

**
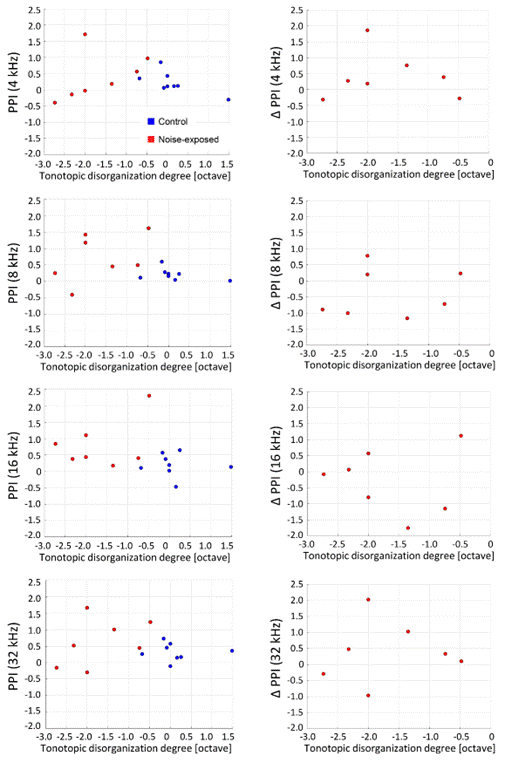
**

**Supplementary Figure S2.** Behavioral metrics of hyperacusis (PPI and ΔPPI) plotted against the tonotopic disorganization degree in the exposed group. Correlation coefficients in PPI with respect to the tonotopic disorganization degree (left column): R=-0.0684 (t-test, p=0.809) at 4 kHz; R=-0.229 (p=0.411) at 8 kHz; R=-0.265 (p=0.341) at 16 kHz; R=-0.0655, p=0.817 at 32 kHz. ΔPPI (right): R=-0.0727 (t-test, p=0.877) at 4 kHz; R=0.143 (p=0.760) at 8 kHz; R=-0.0314 (p=0.947); and R=0.0803 (p=0.864) at 32 kHz.

**
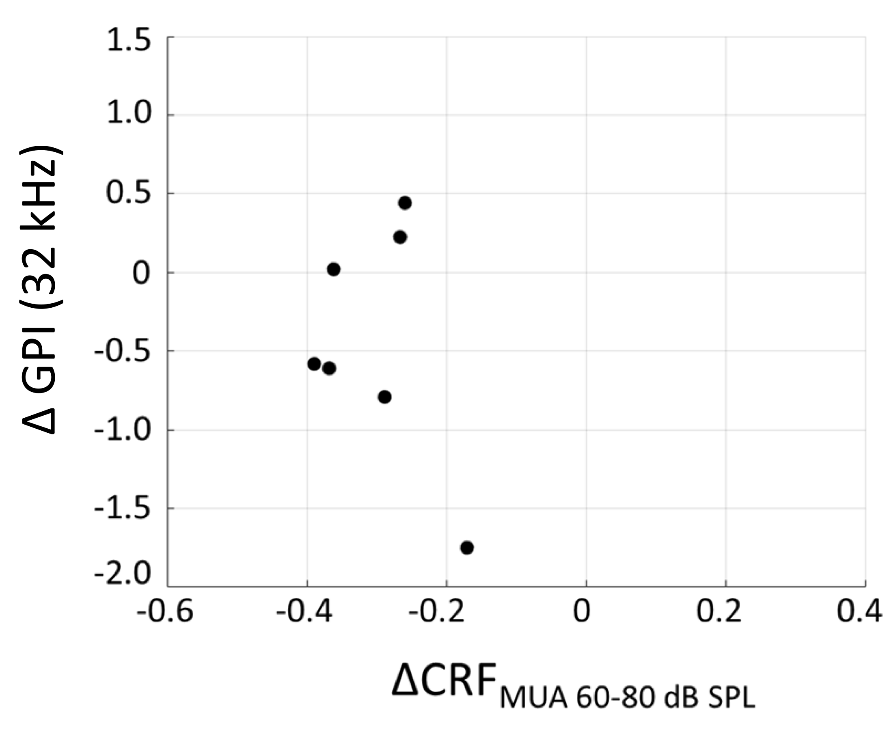
**

**Supplementary Figure S3.** Behavioral metrics of tinnitus (ΔGPI at 32 kHz) plotted against ΔCRFMUA 60-80 dB SPL. Correlation coefficients: R=-0.3454 (t-test, p=0.4480).
